# Cell Responses to Electrical Pulse Stimulation for Anticancer Drug Release

**DOI:** 10.3390/ma12162633

**Published:** 2019-08-19

**Authors:** Anna Puiggalí-Jou, Luis J. del Valle, Carlos Alemán

**Affiliations:** 1 Departament d’Enginyeria Química, EEBE, Universitat Politècnica de Catalunya, C/Eduard Maristany 10–14, Ed. I2, 08019 Barcelona, Spain; 2 Barcelona Research Center for Multiscale Science and Engineering, Universitat Politècnica de Catalunya, Eduard Maristany 10–14, 08019 Barcelona, Spain

**Keywords:** anticancer activity, cell damage, conducting polymer, drug delivery, electrostimulation, nanoparticles, polycaprolactone, polyesters

## Abstract

Electrical stimulation is an attractive approach to tune on-demand drug release in the body as it relies on simple setups and requires typically 1 V or less. Although many studies have been focused on the development of potential smart materials for electrically controlled drug release, as well as on the exploration of different delivery mechanisms, progress in the field is slow because the response of cells exposed to external electrical stimulus is frequently omitted from such investigations. In this work, we monitor the behavior of prostate and breast cancer cells (PC-3 and MCF7, respectively) exposed to electroactive platforms loaded with curcumin, a hydrophobic anticancer drug. These consist in conducting polymer nanoparticles, which release drug molecules by altering their interactions with polymer, and electrospun polyester microfibres that contain electroactive nanoparticles able to alter the porosity of the matrix through an electro-mechanical actuation mechanism. The response of the cells against different operating conditions has been examined considering their viability, metabolism, spreading and shape. Results have allowed us to differentiate the damage induced in the cell by the electrical stimulation from other effects, as for example, the anticancer activity of curcumin and/or the presence of curcumin-loaded nanoparticles or fibres, demonstrating that these kinds of platforms can be effective when the dosage of the drug occurs under restricted conditions.

## 1. Introduction

On-demand local delivery of drug molecules to target tissues provides a means for effective drug dosing, fulfilling requirements for a variety of therapeutic applications while reducing the adverse effects of systemic drug delivery [1,2,3,4]. Recent advances have facilitated the use of various cues, such as UV- and visible-wavelength light, near-infrared (NIR) radiation, magnetic field, ultrasound and electrical stimulation to trigger drug release in vivo from implanted smart materials [1,4,5]. Among them, pulsatile electrically stimulated drug delivery devices have drawn attention not only because they allow repeatable and reliable drug release flux for clinical needs but also because of their simplicity and versatility. Thus, various types of electrically modulated devices for drug release, such as hydrogel [6,7,8,9], nanoparticles [10,11,12,13], membranes [14,15] and fibres [16,17,18,19] have been reported in literature. Besides, electrical stimulation has also been employed in clinics for its potential beneficial effects to revive damaged tissues in the neuromuscular system, reduce the progression of diseases related to the bones such as osteoarthritis and osteonecrosis [20], to reduce pain [21] or to favorably treat Parkinson’s disease [22].

Conducting polymers (CPs), which exhibit characteristics similar to those encountered in metals (i.e., good electrical, magnetic and optical properties) and the outstanding properties of conventional polymers (i.e., flexibility in processing, lightness of weight, and ease of synthesis), are frequently the main component of electrically driven drug-delivery systems. Thus, the excellent redox properties of CP matrices promote the uptake (oxidation) and the expulsion of (reduction) charged drugs, the alteration of the electrostatic forces to facilitate the load or release of charged drugs being typically regulated by applying an external electric field. Besides, the actuation behavior (i.e., expansion and contraction) of CPs can be considered as another important and effective driving force in the drug-delivery process [23,24]. Thus, the actuation experienced by CPs upon oxidation and reduction processes alters the porosity of the polymer matrix, regulating the release of drug molecules, especially of neutral ones. Typically, electrostatic and actuation driving forces coexist to a greater or lesser extent depending on the characteristics of the drug and the CP matrix.

**Scheme 1:**
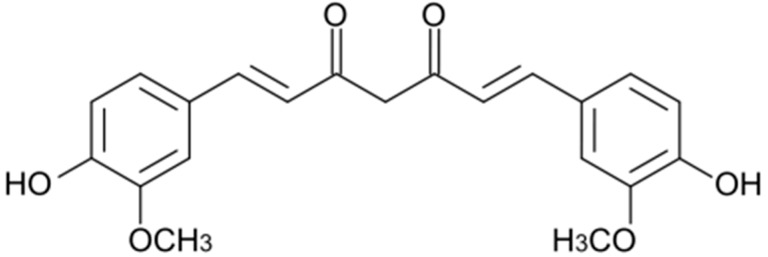
Structure of curcumin (CUR).

Investigations in electrically stimulated polymer devices have been mainly focused to achieve controlled triggered drug delivery and to ascertain the mechanism involved in this process by weighting the electrostatic interactions and actuating properties as driving forces [6,7,8,9,10,11,12,13,14,15,16,17,18,19]. In a recent review, the different mechanisms involved in the release of drugs upon electrical stimuli have been systematically and extensively discussed [25]. Thus, the mechanism depends not only on the redox state of the electrically active material (i.e., they can incorporate or release anionic or cationic molecules on-demand) but also, on the format of the carrier (e.g., films, particles and fibres). However, electrical stimulation also affects the cell growth and geometry, which is usually not taken into account. It is well-known that at the cellular level, electrical stimulation can contribute to cell proliferation [26], migration (electrotaxis) [27], differentiation [28], endocytosis [29] and membrane permeabilization [30]. Particularly, at the intracellular level, changes are produced in the Ca^2+^ entry modulation, in the integrin conformation, induction of plasma membrane depolarization, redistribution of the transmembrane proteins and reorganization of cytoskeletal structure. However, in this work, we investigate the influence of electrically stimulated drug delivery on cells viability and geometry (i.e., cell shape and area) using two different polymeric devices that were successfully used to electrically stimulate the release of curcumin (CUR) [12,16]. The latter is a neutral drug (Scheme 1) with a wide spectrum of medical properties ranging from anti-bacterial, anti-viral, anti-protozoal, anti-fungal, and anti-inflammatory activities to anti-cancer effects [31,32,33]. The two studied devices are the following:

(1) Poly (3,4-ethylenedioxythiophene) nanoparticles (PEDOT-NPs) loaded with CUR, hereafter denoted PEDOT-NPs/CUR [12]. In this case, the delivery of CUR was regulated by controlling the strength of PEDOT···CUR-specific interactions that became weaker when a reductive potential was applied to the loaded PEDOT-NPs. For example, the release measured after 3 min at −0.50 V, −1.0 and −1.25 V was determined to be 9.9%, 30.4% and 38.4%, respectively. According to these experimental observations [12], the proposed mechanism was described as follows:

(PEDOT^*α*^ + *α*DBSA^−^)···*n*CUR + *β*e^−^ → (PEDOT^(*α*−*β*)^ + (*α* − *β*) DBSA^−^)···*m*CUR + *β*DBSA^−^ + (*n* − *m*) CUR

where *n*CUR corresponds to the drug molecules hydrogen bonded to the oxidized PEDOT-NPs, *m*CUR corresponds to the drug molecules that remain hydrogen bonded to the PEDOT-NPs after injecting *β* electrons into the system, applying an external voltage, and *α*+ and (*α* − *β*)+ are the oxidation states of oxidized and reduced PEDOT, respectively (i.e., when PEDOT is completely reduced, *α* = *β*, the charge of the conducting polymer is zero). The electro-regulated release of CUR was found to grow logarithmically with time. For example, when a reduction potential of −1.25 V was applied for a time t, the amount of released CUR increased from 7.1% for t = 30 s to 60.2% for t = 9 min [12].

(2) Electrospun poly (ε-caprolactone) (PCL) microfibres loaded with both PEDOT-NPs and CUR, hereafter denoted PCL/PEDOT-NPs/CUR fibres [16]. In this case, PEDOT-NPs, which were mainly located inside the PCL microfibres, behaved as electro-actuators upon application of well-defined potential pulses, increasing their diameter by ~17% and migrating from inside the PCL matrix to the surface of the microfibres. This electro-mechanical actuation behavior affected the structure of the PCL matrix and promoted the release of curcumin, the latter increasing with the number of pulses [16]. For example, the release of CUR increased from 8% to 30% when the number of square potential pulses of 1 V for 60 s grew from one to five (separated for 5 s).

Considering that the biocompatibility of both PEDOT [34] and PCL [35] is well-known and that the mechanisms for the release of CUR from PEDOT-NPs/CUR and PCL/PEDOT-NPs/CUR were demonstrated, in this study, we provide a new perspective that exclusively analyzes how the operating conditions used for application and regulation of pulsatile electrical stimulation affect the surrounding cells. For this purpose, a dual study comparing two different conditions, without and with electrical stimulation, has been conducted. It is worth noting that, although in some studies the impact of the electric potential on some aspects of cell health has been reported [25], to the best of our knowledge, no systematic study has previously been conducted. Moreover, this is especially true for the release of CUR.

## 2. Materials and Methods

### 2.1. PEDOT-NPs and PEDOT-NPs/CUR

The synthesis of the PEDOT-NPs was conducted by emulsion polymerization in water at 40 °C using 3, 4-ethylenedioxythiophene (EDOT) monomer, sodium dodecylbenzene sulfonate (DBSA) as stabilizer and doping agent simultaneously, and ammonium persulfate (APS) as the oxidizing agent. For PEDOT-NPs/CUR, the drug was loaded in situ during the same polymerization using a 10 mg/mL CUR solution in ethanol.

In brief, 0.0163 g of DBSA was added to a tub filled with 4.5 mL of milli-Q water and the solution was stirred for 1 h at 750 rpm at room temperature. Then, 23.6 mg of EDOT monomer alone (PEDOT-NPs) or with 0.5 mL of the CUR solution in ethanol (PEDOT-NPs/CUR) were added. Again, the resulting solution was stirred for 1 h at 750 rpm at room temperature. Finally, 91.2 mg of APS dissolved in 0.5 mL of milli-Q water was added to the mixture. The reaction was maintained in agitation at 40 °C overnight and protected from light with aluminium foil. No sedimentation was observed after the reaction, indicating a good colloidal stability. Side products and unreacted chemicals were eliminated by a sequence of 3 centrifugations at 11000 rpm for 40 min at 4 °C. The resulting supernatants were decanted and the pellet was re-dispersed in deionized water by using a vortex and a sonic bath (15 min at room temperature). Due to its hydrophobicity, CUR remained in the cores of the surfactant micelles rather than interacting with the medium. The drug loading ratio, expressed as mass of encapsulated drug with respect to the total mass, was 5.9% ± 1.6%.

Cyclic voltammetry (CV) studies were conducted with an Autolab PGSTAT302N galvanostat equipped with the ECD module (Ecochimie, The Netherlands). Measurements were performed on 10 µL of 10 mg/mL NPs solution dried on glassy carbon electrodes (GCE) of diameter = 2 mm. All electrochemical assays were performed using a three-electrode one-compartment cell under a nitrogen atmosphere and at room temperature. The cell was filled with 10 mL of phosphate saline buffer (PBS) solution (pH 7.4) as a supporting electrolyte. Covered or bare GCE was used as the working electrode, platinum as the counter electrode, while an Ag|AgCl electrode containing KCl saturated aqueous solution was the reference electrode (offset potential versus the standard hydrogen electrode, E° = 0.222 V at 25 °C). Oxidation–reduction cycles were registered within the potential range of −0.4 to + 0.8 V at 100 mV/s scan rate.

### 2.2. PCL and PCL/PEDOT-NPs/CUR Fibres

A mixture of PCL, PEDOT-NPs and CUR was prepared as follows for electrospinning: PCL (5.5 g) was dissolved in 32 mL of a mixture of chloroform and acetone 2:1 (v/v). The solution was kept in 37 °C for 24 h under stirring at 100 rpm. PEDOT-NPs (10 mg/mL) and CUR (1.04 mg/mL) were dispersed and dissolved, respectively, in 0.5 mL of ethanol. Finally, 0.2 mL of PEDOT-NPs and CUR solutions were mixed with 1.8 mL of PCL solution and loaded in a 5 mL plastic syringe for delivery through an 18 G × 1.1/2′′ needle at a mass-flow rate of 10 mL/h using an infusion pump. The content of PCL, PEDOT-NPs and CUR in the optimized electrospinning mixture was 15.45% (w/v) of PCL, 0.6 wt% of PEDOT NPs and 0.06 wt% CUR. As a control, fibres of pure PCL were produced using a 17.18% (*w*/*v*) concentration of polymer in 2:1 chloroform:acetone.

The choice of the processing conditions (i.e., distance between the syringe tip and the collector, voltage and the flow rate) were selected on the basis of previous experiments devoted to optimize the morphology of the electrospun microfibres [16]. Thus, the formation of droplets and electrospun beads was completely avoided when microfibres were obtained by applying a voltage of 15 kV and using a needle-tip-collector distance of 15 cm.

### 2.3. Scanning Electron Microscopy (SEM)

Micrographs were obtained using a Focused Ion Beam Zeiss Neon 40 scanning electron microscope operating at 10 kV. Samples were mounted on a double-side adhesive carbon disc and sputter-coated with a thin layer of carbon to prevent sample charging problems. The effective diameter of the nanoparticles and electrospun microfibres was determined from the SEM images using the software SmartTIFF (v1.0.1.2.). In order to visualize cells, before the carbon coating, samples with cells were fixed in a 2.5% formaldehyde PBS solution (pH = 7.2) overnight at 4 °C. Then, they were dehydrated by washing in an alcohol battery (30°; 50°; 70°; 90°; 95°; and 100°) at 4 °C for 15 min per wash. Finally, samples were air-dried and sputter-coated with carbon.

### 2.4. 3D Cell Culture and Cell Morphology by Transmission Electron Microscopy (TEM) and Confocal Microscopy

PC-3 (human prostate cancer cell line) and MCF7 (human breast cancer cell line) cells, which are frequently used in cancer research and drug development, were used for the experiments. Both PC-3 and MCF7 cell lines (were obtained from ECACC (European Collection of Cell Culture, London, UK). The previously described CUR delivery systems, as well as their corresponding controls (i.e., PEDOT-NPs and PCL microfibres), were sterilized with an UV lamp for 30 min on both sides and attached with non-toxic silicon to the flat bottom of the wells in a 24-well/plate. Cells were seeded at a density of 40000 cells/mL in Advanced Dulbecco’s Modified Eagle’s Medium (DMEM) supplemented with 5% fetal bovine serum, 1% penicillin/streptomycin and 4 mM L-glutamine and incubated overnight at 37 °C and 5% of CO_2_. The next day, cells were washed gently with PBS and adhesion was evaluated by MTT [3-(4,5-dimethylthiazol-2-yl)-2,5-diphe-nyltetra-zolium] which was performed according to manufacturer instructions. Assays with *n* = 3 were repeated two times independently. The 2,2′-azino-bis 3-ethylbenzthiazoline-6-sulfonic acid (ABTS) method was to measure the radical scavenging activity.

The morphology of the cells incubated with the PEDOT-NPs was observed by TEM. Images were obtained with a Philips TECNAI 10 electron microscope operating at 100 kV. Bright field micrographs were taken with a SIS MegaView II digital camera). A total of 5 × 10^5^ cells/mL were cultured for 24 h in a sterile T-25 flask, and PEDOT NPs (25 µg/mL) were added for another 24 h. After incubation, cells were washed with 0.1 M phosphate-buffered saline solution (PBS), trypsinized with 0.25% Trypsin-EDTA (Gibco, Langley, OK, USA), counted and 1 million cells collected in an Eppendorf. Then, they were prefixed with a modified Karnovsky’s fixative (mixed with 2% paraformaldehyde and 2% glutaraldehyde in 0.05 M PBS buffer) at 4 °C for 2 h. After being washed three times with 0.1 M PBS, cells were post-fixed with 1% osmium tetraoxide at 4 °C for 2 h, washed 3 times with milli-Q water and stained with 0.5% uranyl acetate at 4 °C overnight while being protected from light. Afterwards, dehydration was conducted through a graded series of 30%, 50%, 70%, 80%, 90%, and 100% ethanol (15 min each) and then slowly embedded within the resin by a series of 2:1, 1:1 and 0:1 of propylene oxide: spurr’s resin (30 min each). Resin blocks were cured at 65 °C for 2 days and sectioned by ultramicrotomy. Before analyses, the sections were stained with 2% uranyl acetate and Reynolds’ lead citrate.

Confocal microscopy imaging was performed using an Axio Observer Z1 fluorescence microscope (Carl Zeiss) confocal laser scanning microscope with a 10× and 40× objectives. Morphology studies were performed with ImageJ software. Cells were fixed and stained for nucleus and F-actin on day 1. After 24 h, cells were washed with PBS and fixed with 2.5% paraformaldehyde in PBS for 40 min at room temperature. Later on, samples were washed 3 times for 5 min each with PBS and permeabilized with 0.05% (w/v) triton X-100 in PBS for 20 min under agitation. After this, unspecific sites were blocked with a solution containing 1% bovine serum albumin, 22.52 mg/mL glycine and 0.1% Tween-20 in PBS for 30 min. F-actin filaments were stained with phalloidin atto-488 (Stock solution 10 nmol/500 uL methanol) used with a 1/50 dilution in PBS for 60 min at room temperature under agitation. Again, samples were washed 3 times for 5 min each with PBS. Finally, the cell nucleus was stained with bis-Benzimide H33258 (Stock solution 2 mM) employed at 1/100 dilution in PBS for a duration of 30 min under soft constant agitation and mounted onto the glass slides. Samples were protected from light and kept at 4 °C before use.

Each data point corresponds to the average of three samples and the error bars refer to the respective standard deviation. The Greek letter in the column indicates a significant difference (*p* < 0.05) when one-way ANOVA and Turkey’s multiple comparison tests have been applied.

## 3. Results and Discussion

It is often challenging to apply electrical stimuli onto monolayer cell cultures. Herein, cells have been seeded onto the anode surface and, initially, our efforts have been devoted to identify the optimal potential difference for therapies based on the controlled release of drugs with the intention of not damaging healthy tissues. Since CUR presents anticancer activity, all the assays have been conducted using prostate and breast cancer cell lines (PC-3 and MCF7, respectively). In particular, voltage-induced cell death assays have been carried out using PC-3 cells seeded on a simple metal substrate that is a representative working electrode among those used for bioelectrical stimulation therapies. Currently, there is a considerable number of materials (e.g., carbon, platinum, gold, titanium, stainless steel and indium tin oxide, among others) that successfully work as potential biomedical electrodes [36]. Among them, due to its inertness, electrochemical stability and corrosion resistance, the most employed is platinum. Nevertheless, it is limited by its poor mechanical stability and high cost. Herein, we have chosen stainless steel (AISI 316) because for short-duration pulsatile electrical stimulation protocols based on the application of low intensities, there is no risk of decomposition of the electrode and steel is more resistant to mechanical failures.

**Figure 1:**
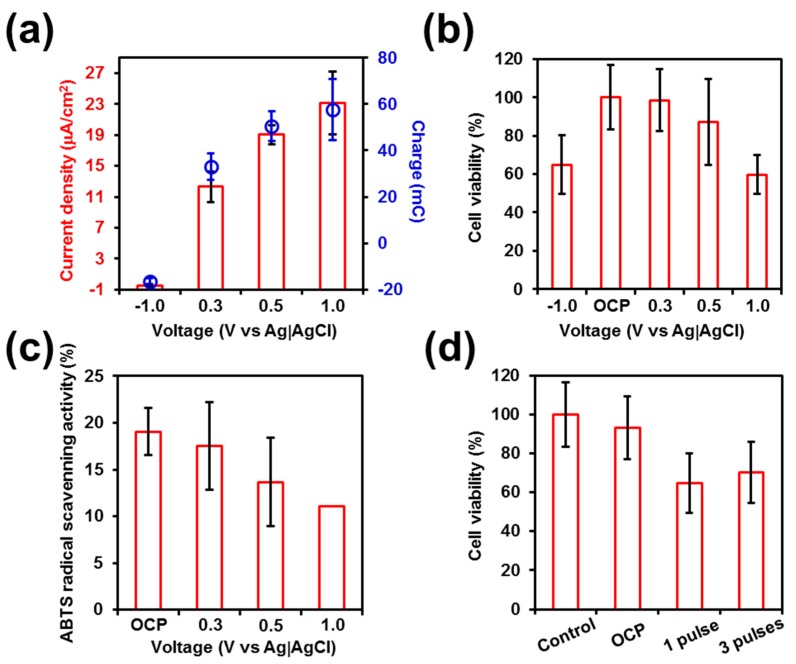
(**a**) Relation between the applied voltage and both the current density (bars) and the accumulated charge (circles). (**b**) Cell viability and (**c**) radical scavenging activity after applying a voltage pulse for 1 min. (**d**) Cell viability after application of one and three pulses of 1.0 V for 1 min (Control and OCP refer to non-stimulated and open circuit potential, respectively). In all cases, cells were cultured on stainless pins (0.5 × 1.0 cm^2^) within a tissue well-plate (20 × 10^3^ cells/well) for 24 h. After this time, pins were used as working electrodes and pulses were applied at the desired voltage for 1 min. Finally, the pins were placed in a new tissue well-plate for 24 h before the MTT and ABTS assays were performed. Each data point corresponds to the average of three samples and the error bars refer to the respective standard deviation.

The response of PC-3 cells seeded on stainless steel pins to electrical stimulation was studied by varying separately the following operating conditions: the voltage, the number of pulses applied, and the duration of such pulses (Figure 1). Negative voltages typically result in an enhancement of reduction reactions, while positive voltages cause an increase in oxidation reactions and higher ion release from metallic surfaces, with the main drawback in this case being the dissolution of iron from the stainless steel substrate [36,37]. Furthermore, there is an increase in reactive oxygen species (ROS) products on the electrode surface, which can cause oxidative cellular stress. Initially, the response of cells cultured for 24 h onto the steel pins towards voltages of −1.0, 0.3, 0.5 and 1.0 V, which were applied for 1 min, was examined.

Figure 1a shows the implication of such voltage treatments in terms of current density, which ranges from −0.5 (−1.0 V) to 23 μA/cm^2^ (1.0 V), and accumulated charge, which varies from −17 (−1.0 V) to 58 mC (1.0 V). Thus, the effects of those voltages were evaluated 24 h after their application, determining the cell viability. Figure 1b shows that the cell viability underwent a significant reduction when voltages of 1.0 and −1.0 V were employed, diminishing in both cases to ~60%. Amazingly, the electro-regulated release of CUR from PEDOT-NPs/CUR started at −0.5 V with 10%, and grew to 30% and 38% when the reductive voltage decreased to −1.0 and −1.2 V, respectively [12]. Similarly, the maximum effectivity for the release of CUR from PCL/PEDOT-NPs/CUR was achieved when a voltage of 1.0 V was applied [16], which as shown in Figure 1, severely affects the cell viability. These observations indicate that the on-demand drug release by electrical stimulation should consist of a balance between the effectivity in the release kinetics and the cell health.

The radical scavenging activity of cells seeded onto the stainless steel was halved along with cell viability when a voltage of 1.0 V was applied (Figure 1a–c). It is well-known that oxygen molecules can generate hazardous products called ROS during reactions occurring in the intracellular space. Hence, cells have an antioxidant defense system to keep free radical formation controlled. However, in our case, this defense was not minored after the use of 1.0 V for 1 min since its decrease was proportional to the decrease in cell viability. Therefore, this observation let us conclude that cells might be damaged following another underlying mechanism or the combination of various mechanisms. We hypothesize that this effect may be due to the associated nano-toxicology of the stimulated stainless steel where the cells are seeded.

The influence of the number of pulses was evaluated by determining the cell viability after applying one or three pulses of 1.0 V for 1 min (Figure 1d). As it can be seen, significant differences were observed between non-stimulated and electro-stimulated cells, the viability being ~30% lower for the latter than for the former. However, no appreciable statistical difference was observed between one and three potential pulses, suggesting that the damage caused by the accumulated charge and the current density occurs after the first pulse. Overall, findings reported in Figure 1 suggest that reductive and moderately oxidative voltages do not cause important alterations in cell viability and metabolism while, in contrast, application of a potential voltage as high as 1.0 V is clearly pernicious. This point is crucial for the utilization of electrically stimulated drug-delivery devices since it would not be easy to differentiate whether the harmfulness is caused by the applied voltage or by the released drug itself.

**Figure 2:**
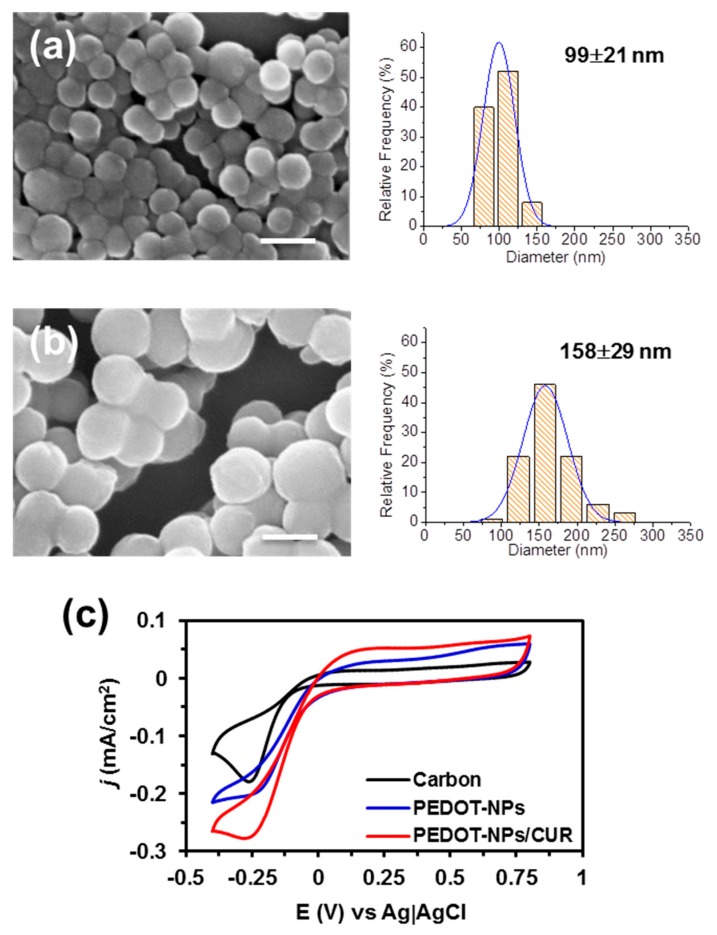
SEM images of (**a**) Poly (3,4-ethylenedioxythiophene) nanoparticles (PEDOT-NPs) and (**b**) PEDOT-NPs/CUR (Scale bar: 200 nm). Effective diameter histograms derived from SEM measurements and average values are also displayed. (**c**) Cyclic voltammograms of the carbon anode coated with 10 uL PEDOT-NPs and PEDOT-NPs/CUR (10 mg/mL).

Taking the results displayed in Figure 1 into consideration, PC-3 cells were incubated onto PEDOT-NPs and PEDOT-NPs/CUR. The diameter of the PEDOT-NPs and PEDOT-NPs/CUR, was 99 ± 21 and 158 ± 29 nm, respectively, as determined by SEM (Figure 2). Electroactivity and electrostability of the PEDOT-NPs and PEDOT-NPs/CUR were evaluated by means of cyclic voltammograms. The voltammogram area increases when the electrodes are coated with PEDOT-NPs and PEDOT-NPs/CUR (Figure 2c), with the latter showing the oxidation peak of the drug at 0.3 V.

These materials present high electroestability because, after 10 consecutive cycles, its electroactivity loss was lower than 15%. Electrical stimulation experiments were undertaken using voltages of 0.5 V and −0.5 V. The cytoxicity of PEDOT-NPs and PEDOT-NPs/CUR for PC-3 cells, expressed as half-maximal cytotoxic concentration (CC50) is around 500 mg/mL and 100 µg/mL, respectively, as reported in previous work [12].

**Figure 3:**
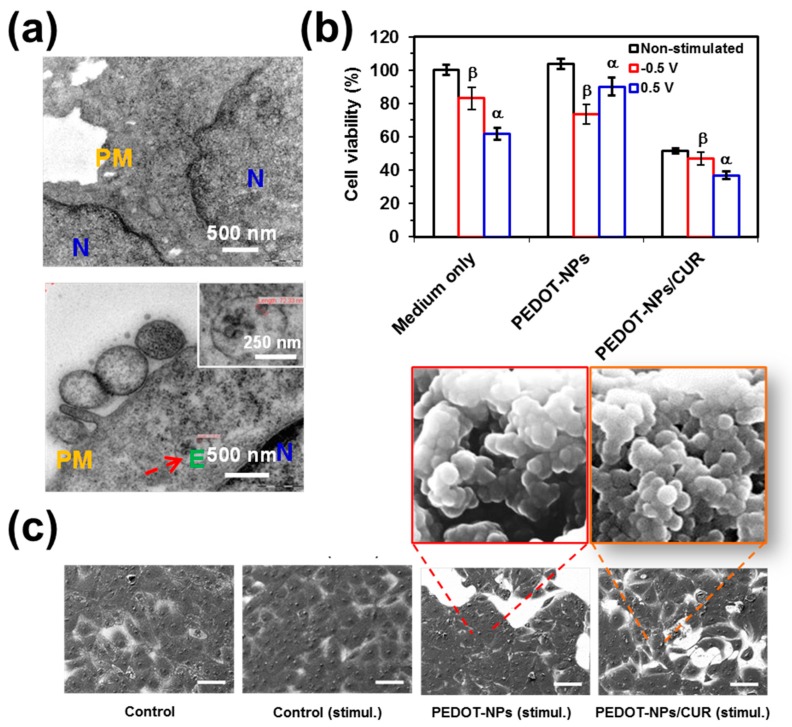
(**a**) TEM images of cells incubated without and with PEDOT-NPs (25 mg/mL) for 24 h (top and bottom, respectively). Read arrows indicate the PEDOT-NPs. N, E and PM refer to nucleus, endosome and plasma membrane. (**b**) Relative cell viability of PC-3 cells cultured on stainless steel pins (control), PEDOT-NPs and PEDOT-NPs/CUR non-stimulated and after one pulse of 30 s of 0.5 V or −0.5 V. The data points correspond to the average of three samples and the error bars refer to the respective standard deviation. Greek letters in the columns indicate a significant difference (*p* < 0.05) when one-way ANOVA and Turkey’s multiple comparison tests have been applied. (**c**) SEM micrographs (scale bar: 100 µm) of cell growth onto the non-stimulated control and electro-stimulated (one pulse of 30 s of 0.5 V) control, PEDOT-NPs and PEDOT-NPs/CUR. Magnifications (scale bar: 100 nm) represent the PEDOT-NPs and PEDOT-NPs/CUR that were not endocytosed and remained on the cell surface.

TEM micrographs of non-treated cells and cell incubated with PEDOT-NPs are compared in Figure 3a. As it can be seen, cells are able to endocite PEDOT NPs. Accordingly, herein, a concentration below CC_50_ values (e.g., 50 µg/mL) was considered to examine the cell viability after electrical stimulation. Figure 3b compares the viabilities of cells cultured on PEDOT-NPs, PEDOT-NPs/CUR and steel (control) after electrostimulation (i.e., the voltage was applied after 24 h of cell culture and the viabilities were determined 24 h after applying the potential) with those of non-stimulated control samples (i.e., viabilities determined after 48 h of cell culture). Results reveal that concentrations of PEDOT-NPs/CUR, which were not harmful in absence of electrical stimuli, caused a drastic reduction in terms of cell viability after electro-stimulation. More specifically, the cell viability was halved after applying a voltage of 0.5 or −0.5 V. In contrast, the cell viability was only reduced to 75% when steel and PEDOT-NPs were electrically stimulated. These observations indicate that, as was expected, CUR was delivered by electrical stimulation but also that the drug preserves the anticancer activity.

Cell morphologies were characterized by SEM (Figure 3c). A monolayer of cells was observed on the stainless steel pins used as a control, independently of the presence of electrical stimulus. Similarly, cells seeded on PEDOT-NPs presented a spread appearance as well, even after the application of the voltage. Instead, electrically stimulated PEDOT-NPs/CUR underwent a drastic reduction of both the area covered by cells and the density of contacts between cells. These observations are fully consistent with cell viabilities, indicating that, despite its anti-cancer activity and other beneficial therapeutic properties, CUR is not a completely safe drug and exhibits some cytotoxicity.

**Figure 4:**
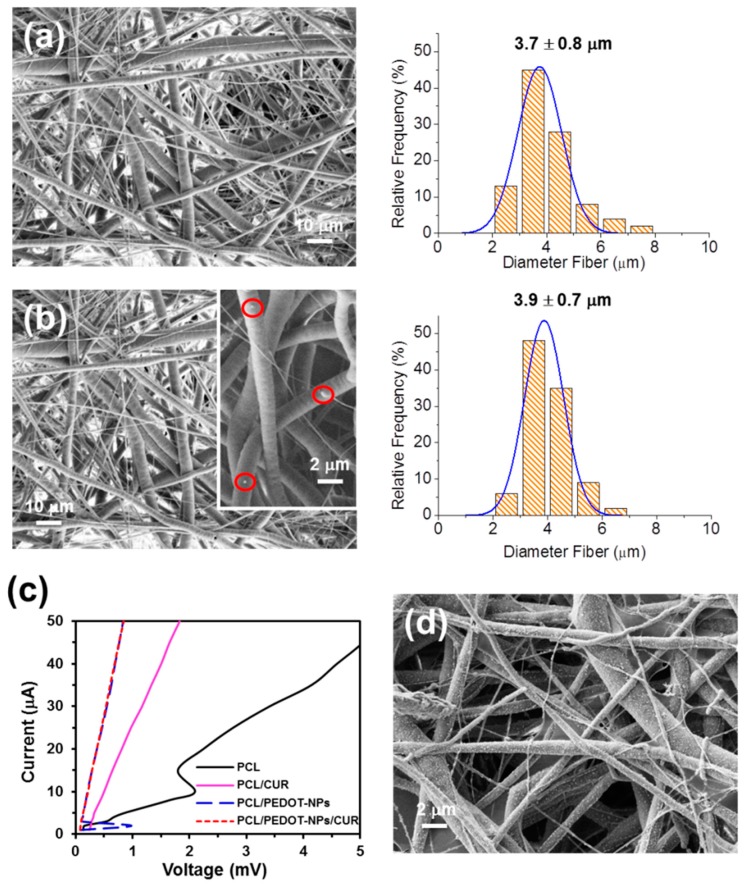
SEM micrographs of electrospun (**a**) poly (ε-caprolactone) (PCL) and (**b**) PCL/PEDOT-NPs/CUR fibrous mats before electrical stimulation. Effective-diameter histograms derived from SEM measurements and average values are also displayed. The inset in (**b**) shows a few PEDOT-NPs (red circles) on the surface of the fibres. (**c**) Typical I–V curves obtained using different applied voltages. (**d**) Representative SEM micrograph of a PCL/PEDOT-NPs/CUR fibrous mat after applying a voltage of 0.5 V for 1 min.

After this, the impact of the electric field on PC-3 and MCF7 human cancer cells seeded onto PCL/PEDOT-NPs/CUR fibrous mats was evaluated and observations were correlated with the amount of delivered CUR. For this purpose, PCL (blank) and PCL/PEDOT-NPs/CUR (target) microfibres were prepared. The contact angles of PCL and PCL/PEDOT-NPs/CUR were reported to be 128° ± 4° and 112° ± 7°, respectively [16]. Figure 4a,b display representative SEM micrographs of PCL and PCL/PEDOT-NPs/CUR fibres, respectively. Electrospun PCL mats are formed by a random distribution of homogeneous microfibres with a diameter of 3.7 ± 0.8 µm and a smooth surface. Instead, PCL/PEDOT-NPs/CUR microfibres, which present a diameter of 3.9 ± 0.7 µm, were proven to exhibit individually segregated PEDOT-NPs. These were mainly distributed inside the polyester matrix, even though a few PEDOT-NPs were also located at the surface of the polyester matrix, as is illustrated in Figure 4b (inset). The conductivity of the different mats was evaluated using a four-proves machine and by comparing the slopes of the current-voltage (I-V) curves (Figure 4c). The conductivity decreases as follows PCL/PEDOT-NPs~PCL/PEDOT-NPs/CUR > PCL/CUR > PCL. These results indicate that the incorporation of PEDOT-NPs, as suspected, increased the conductivity of the material, making it ideal for promoting an electrostimulated drug delivery.

The release mechanism from PCL/PEDOT-NPs/CUR is based on the events caused in PEDOT-NPs by electrical stimulation (i.e., conformational movements, electrostatic repulsions and compositional variations through the incorporation of hydrated anions), which induce changes in their volume [16]. Thus, the mechanical energy associated to such a volume increment is used to alter the structure of PCL microfibres, resulting in the movement of PCL molecules, thereby generating macroscopic CUR release. In addition, electrical stimulation promotes the appearance of many PEDOT-NPs at the surface of the microfibres. This is illustrated in the SEM micrograph displayed in Figure 4d, which shows the large amount of PEDOT NPs after applying a voltage of 0.5 V for 1 min, confirming that most of the PEDOT-NPs were initially located inside the PCL matrix.

**Figure 5:**
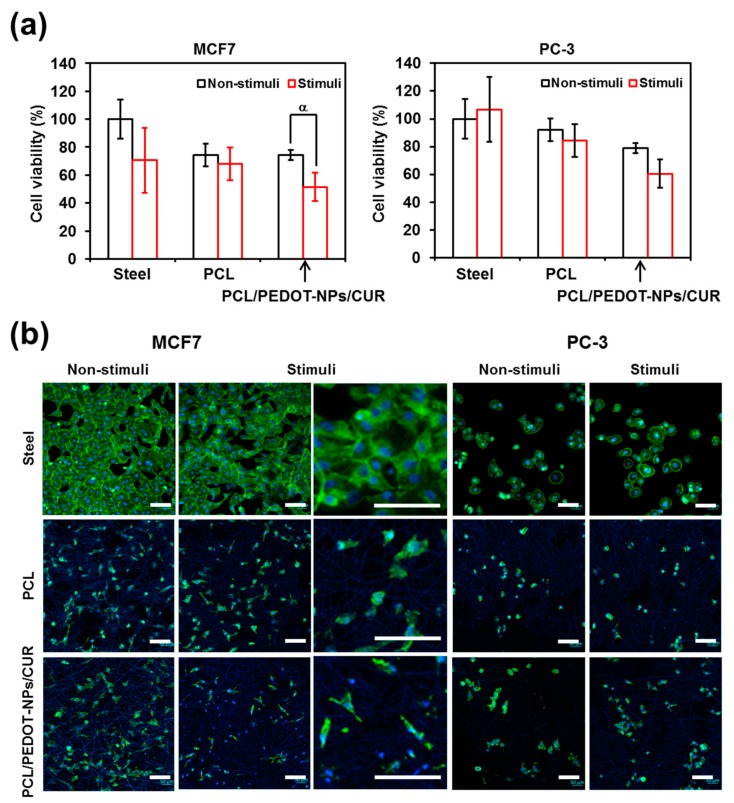
(**a**) Relative cell viability of PC-3 and MCF7 without and with electrical stimuli (1 pulse of 0.5 V for 1 min). (**b**) Morphology of cells cultured onto stainless steel pins and both PCL and PCL/PEDOT-NPs/CUR fibrous mats without and with electrical stimuli (1 pulse of 0.5 V for 1 min). Confocal microscopy micrographs recorded after 48 h of cell culture (non-stimuli) or 24 h after applying the electrical stimuli, which in turn, was carried out 24 h after starting the cell culture. Cells were stained for the nucleus in blue (Hoechst) and F-actin in green (Phalloidin Atto 488). Magnified images are also displayed for MCF7 cells cultured with electrical stimuli. In all cases, scale bar = 100 μm. Each data point in (**a**) corresponds to the average of three samples and the error bars refer to the respective standard deviation. The Greek letter in the column indicates a significant difference (*p* < 0.05) when one-way ANOVA and Turkey’s multiple comparison tests have been applied.

Electrical stimuli, which consisted of 1 pulse of 0.5 V for 1 min each, were applied to bare stainless steel pins (control) and steel pins coated with electrospun PCL or PCL/PEDOT-NPs/CUR fibrous mats. Figure 5a compares cell viabilities for the different substrates. As is shown, the viability of cells seeded onto PCL is higher for the PC-3 line than for the MCF7 line, indicating a dependence on the characteristics and morphology of cells, which directly affect their affinity towards PCL fibres. On the other hand, CUR released from PCL/PEDOT-NPs/CUR by electrical stimulation considerably influences the survival of cancer cells. As was expected, differences in the viability of cells subjected or not to electrical stimuli were not significant for the control and blank substrates, independently of the cell line. Thus, the applied potential was not high enough to damage the cells (Figure 1b) and no anticancer drug to induce cell death was loaded into these substrates. In contrast, significant differences were encountered in the case of the cells seeded onto PCL/PEDOT-NPs/CUR matrices, reflecting that the regulated CUR release diminished the viability of cancer cells. It is well known that CUR could induce apoptosis in most, but not all, cancer cell lines by inducing changes in the cell membrane potential [38]. According to the literature, MCF7 tumor cells are very sensitive to the presence of CUR [39]. For the case of PC-3 cells, it was reported that CUR affects the proliferation (anti-proliferative property) but the induced apoptosis is lower than for MCF7 cells [40]. These results show that when the electrical stimulation is carried out in a controlled manner, so that the operational parameters do not damage the cells, the effects produced by the released drug correspond to those desired.

Cell spreading is mainly governed by traction forces exerted by cytoskeletal fibres. F-actin, which is the predominant component of the cytoskeletal machinery, was visualized together with the cell nuclei by confocal microscopy. Figure 5b compares representative images of PC-3 and MCF7 cells cultured on steel pins and both PCL and PCL/PEDOT-NPs/CUR fibrous mats that were not perturbed during 48 h (non-stimulated) and that were electro-stimulated by applying 1 pulse of 0.5 V for 1 min just after 24 h of culture and, subsequently, remained unperturbed until reaching 48 h. As was expected, colonization and spreading of these substrates are consistent with the cell viabilities displayed in Figure 5a. Thus, the lowest cell spreading, which corresponds to the electrically stimulated PCL/PEDOT-NPs/CUR, has been associated with the activity of the released drug. In contrast, no significant difference is apparently observed between cells colonizing non-stimulated and stimulated control and blank substrates.

**Figure 6:**
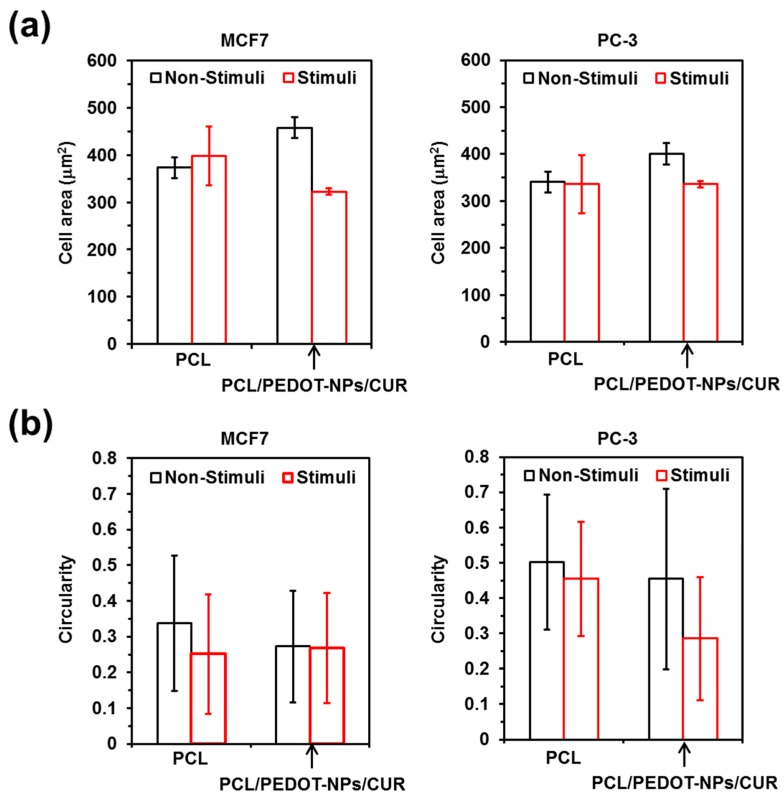
Quantification of (**a**) cell area and (**b**) cell circularity of MCF7 and PC-3 cells cultured onto both PCL and PCL/PEDOT-NPs/CUR fibrous mats without and with electrical stimuli (1 pulse of 0.5 V for 1 min). Each data point corresponds to the average of three samples and the error bars refer to the respective standard deviation.

In order to provide more insights about the changes induced by electrical stimulation on PC-3 and MCF7 cells, both the cell area and cell circularity were analysed with the ImageJ software (Figure 6). These analyses were conducted using representative confocal microscopy micrographs of cells spread onto PCL and PCL/PEDOT-NPs/CUR fibrous mats. Regarding cell area measures, results indicate that the major observable difference between the non-stimulated and the electro-stimulated substrates corresponds to the decrease in the cell area for the PCL/PEDOT-NPs/CUR matrices. In contrast, this change is not detected for cells cultured onto PCL scaffolds (Figure 6a). Considering the results reported in Figure 1, Figure 3b and Figure 4a, the reduction in the area of cells in contact with electro-stimulated PCL/PEDOT-NPs/CUR microfibres with respect to non-stimulated ones were mainly attributed to the anticancer activity of the drug and/or the simple presence of CUR-loaded fibres rather than to the operating conditions employed for the electrical stimulation.

On the other hand, the influence of electrical stimulation on cell circularity is unmeaningful, the main features displayed in Figure 5b are due to the influence exerted by the fibrous substrates on cell lines with different stiffness. Thus, although MCF7 epithelial-like cells usually present high circularity with values ranging from 0.7–0.9 [41], cells cultured onto fibrous mats become elongated, showing circularities of ~0.3. This loss of roundness is consistent with the high deformability of MCF7 cells, which has been recently reported to exhibit significant shear-induced heterogeneous deformation [42]. More specifically, biophysical analyses of fluid shear stress in a microfluidic device that mimicked the hemodynamic conditions of the blood stream, showed a quick significant reduction in circularity for MCF7 cells. On the other hand, the response of PC-3 cells to the fibrous substrates was less pronounced than that of MCF7 cells. Thus, the cell circularity of PC3 was ~0.5, suggesting a lower deformability than MCF7 cells.

## 4. Conclusions

In summary, we have compared the behavior of CUR-loaded PEDOT-NPs and PLC/PEDOT-NPs microfibres without and with electro-stimulation to preliminarily evaluate the effect of these drug-delivery systems. Results show that these devices can be used as a reservoir of CUR, which can be released upon electrical pulse stimulation. We also demonstrated that the response of prostate and breast tumor cells (PC-3 and MCF7, respectively) exposed to such CUR-loaded electroactive platforms depends on the operating conditions used for electro-stimulation (i.e., magnitude of the voltage, number of pulses, time of each pulse, etc.). For PEDOT-NPs, the response of the cells was appropriated when pulses of 30 s of 0.5 V or −0.5 V were applied. In the case of PCL/PEDOT-NPs, which is based on an electro-actuation mechanism, the duration of the 0.5 V pulses must be increased to 1 min. Thus, controlled electrical stimulation restricting the operational parameters does not damage the cells during the CUR release process. However, the utilization of higher potentials to accelerate the drug release kinetics is harmful, causing a drastic reduction in the cell viability. Taken together, the results indicate that the studied platforms can be electro-stimulated without significant alteration of the cells’ health.
